# Laboratory demonstration of the vertical transmission of Rift Valley fever virus by *Culex tarsalis* mosquitoes

**DOI:** 10.1371/journal.pntd.0009273

**Published:** 2021-03-22

**Authors:** Nicholas A. Bergren, Erin M. Borland, Daniel A. Hartman, Rebekah C. Kading

**Affiliations:** Department of Microbiology, Immunology, and Pathology, Colorado State University, Fort Collins, Colorado; Connecticut Agricultural Experiment Station, UNITED STATES

## Abstract

Rift Valley fever virus (RVFV) is a mosquito-transmitted virus with proven ability to emerge into naïve geographic areas. Limited field evidence suggests that RVFV is transmitted vertically from parent mosquito to offspring, but until now this mechanism has not been confirmed in the laboratory. Furthermore, this transmission mechanism has allowed for the prediction of RVFV epizootics based on rainfall patterns collected from satellite information. However, in spite of the relevance to the initiation of epizootic events, laboratory confirmation of vertical transmission has remained an elusive research aim for thirty-five years. Herein we present preliminary evidence of the vertical transmission of RVFV by *Culex tarsalis* mosquitoes after oral exposure to RVFV. Progeny from three successive gonotrophic cycles were reared to adults, with infectious RVFV confirmed in each developmental stage. Virus was detected in ovarian tissues of parental mosquitoes 7 days after imbibing an infectious bloodmeal. Infection was confirmed in progeny as early as the first gonotrophic cycle, with infection rates ranging from 2.0–10.0%. Virus titers among progeny were low, which may indicate a host mechanism suppressing replication.

## Author summary

Rift Valley fever virus (RVFV) represents a significant threat in terms of its ability to emerge into naïve geographic areas. Furthermore, RVFV represents a global public health risk due to the ability of many mosquito species to transmit the virus and the ease with which the virus can be transported due to increased globalization. The vertical transmission of RVFV by mosquitoes has long been accepted by the research community due to limited field evidence. However, laboratory confirmation of vertical transmission has remained elusive for thirty-five years. We present the first laboratory evidence of vertical transmission of RVFV in the susceptible North American vector, *Culex tarsalis*. We present two studies that clearly show 1) the accumulation of RVFV antigen in the ovaries of infected mosquitoes and 2) the transmission of RVFV from parent to offspring immediately following an infectious blood meal.

## Introduction

Rift Valley fever virus (RVFV) (order: *Bunyavirales*; family: *Phenuiviridae*; genus: *Phlebovirus*) is an emerging, mosquito-transmitted virus endemic to sub-Saharan Africa [1]. Outbreaks are episodic and impose a significant economic, veterinary, and public health burden. Both livestock animals and humans are susceptible to infection. RVFV mortality rates among adult ruminant livestock ranges from 10% to 20%. Additionally, RVFV epizootics are characterized by abortion storms with neonatal mortality rates approaching 100% [2]. Infection in humans typically results in febrile illness but can progress to more severe illness in 1% to 2% affected individuals with a case fatality rate between 10% and 20% [3,4]. In 1997–1998, a large transboundary outbreak of RVFV occurred in Africa, resulting in 90,000 human infections and the loss of approximately 100,000 domestic animals [5]. The outbreak also elicited a ban on livestock exports, which caused significant economic hardship for the region [6]. RVFV also poses significant global emergence potential as evidenced by large and sustained outbreaks in Egypt and the Arabian Peninsula [7–9]. Furthermore, much work has been conducted demonstrating that RVFV could invade and establish itself in North America because of frequent travel from endemic areas, presence of susceptible mosquito vectors, and US agricultural practices such as intensive livestock production [10–16].

Understanding the transmission mechanisms by which arboviruses maintain themselves in nature is critical for assessing their potential risk for emergence and establishment in new places. Arbovirus maintenance is generally perpetuated via horizontal transmission cycles between arthropod vectors and vertebrate hosts. However, some arboviruses have also evolved a means of vertical transmission whereby the virus is transmitted from parental arthropods directly to their offspring. Vertical transmission has been demonstrated among the bunyaviruses, flaviviruses, and alphaviruses indicating that this is a relatively common and convergent evolutionary strategy for virus persistence in the presence of ecological conditions that are not conducive to classical horizontal transmission [17,18].

The mechanisms of RVFV persistence through interepidemic periods remains not fully characterized. RVFV is has the ability to infect and transmit among a wide variety of vectors, which can be classified into two categories: “reservoir/maintenance”, which include certain *Aedes* species (spp.) mosquitoes, and “epidemic/amplifying”, characterized by *Culex* spp. mosquitoes [19]. RVFV relies, at least in part, on vertical transmission in the “reservoir/maintenance” mosquito vector to potentiate itself between epizootic events, however, evidence of this mode of transmission is limited to a few virus isolations from adult mosquitoes reared from field-collected larvae and antigen accumulation in mosquito ovaries [20,21]. Endemic transmission does also occur in between epizootic events, although at low levels [22]. Furthermore, RVFV epizootics have been correlated with periods of abnormally high rainfall and this pattern has been further correlated with El Niño/Southern Oscillation events, providing the capability to identify environmental conditions favorable to supporting an outbreak [23,24]. Although vertical transmission of RVFV has been generally accepted by the scientific community as a mechanism for ecological persistence, to date, this phenomenon has not been demonstrated in a laboratory environment. Understanding the potential impact of vertical transmission on RVFV establishment if this virus is introduced to new geographic areas including North America is critical, as it will provide insight into vector control, mitigation efforts, and entomological surveillance in the midst of an outbreak. Herein, we present three preliminary experiments that demonstrate the vertical transmission of RVFV in a North American mosquito vector *Culex* (*Cux*.) *tarsalis* Coquillett, a vector that has been shown in the laboratory to have the potential to transmit RVFV very efficiently [13].

## Materials and methods

### Ethics statement

All experiments were approved by CSU’s Institutional Biosafety Committee under protocol number 16-078B. Experiments were also conducted in accordance with the CDC and USDA regulations governing the utilization of Select Agents and Toxins.

### Mosquitoes

All *Cx*. *tarsalis* mosquitoes used in these experiments are from the Kern National Wildlife Refuge (KNWR) colony. The colony was established in July 1953 from mosquitoes collected from the Kern National Wildlife Refuge, Kern County, California, USA [25].

### Virus stocks and infectious and non-infectious bloodmeals

Stocks of RVFV (Kenya 128B-15) were generated by infecting Vero cells (ATCC CCL-81, American Type Culture Collection) at a MOI of 0.01 and incubating for 72 hours in a humidified incubator set to 37°C with 5% CO_2_. DMEM supplemented with 2% FBS and Penicillin/Streptomycin was used for virus propagation. Virus for infectious bloodmeals was generated from stock virus in the same manner. At 72 hours post-infection, infectious cell culture supernatant was clarified by centrifugation at 7,000 x g for 10 minutes and was added to defibrinated calf blood (Colorado Serum Company) at a 1:1 ratio. Non-infectious blood meals consisted solely of defibrinated calf blood diluted 1:1 with DMEM, supplemented as described above.

Blood meals were loaded into Hemotek membrane feeders covered in collagen membrane (Hemotek Ltd, Lancashire, United Kingdom) and were warmed to 37°C. Mosquitoes were fed for 2 hours. After feeding, mosquitoes were anesthetized by holding at 4°C until immobilized and were sorted in petri dishes on ice; engorged mosquitoes were placed in new containers and the rest were discarded. Aliquots of infectious bloodmeals were held at the same conditions for the duration of the infectious blood meal, and then were back titrated via plaque assay as previously described to confirm that the target blood meal viral titer was achieved [26].

### Study 1: Detection of RVFV antigen in mosquitoes receiving infectious blood meal

Artificial blood meals with RVFV (7.3 log_10_ PFU/mL) were provided to *Cx*. *tarsalis* females between 5–8 days post-emergence as described above. After feeding, engorged mosquitoes were incubated at 28°C with a 16:8 light:dark cycle and 70% relative humidity for 7 days. The protocol used for fixing and staining of paraffin-embedded sections of mosquitoes was described previously by Kading et al. [27]. Briefly, female mosquitoes used in immunofluorescence assays were injected with 360nl of freshly prepared 4% paraformaldehyde (Electron Microscopy Products) with a Nanoject III (Drummond) and submerged in 4% paraformaldehyde for 24 hours. After 24 hours, samples were transferred to 70% ethanol. Inactivation assays were conducted on 10% of samples to verify inactivation prior to transfer out of BSL3 conditions as per institution-approved inactivation requirements [27,28]. Sectioning was conducted by Colorado HistoPrep (Fort Collins, CO). Mosquito sections were stained with a mouse anti-RVFV polyclonal antibody diluted to 1:1024. Goat anti-mouse Alexa Fluor 488 (Invitrogen) diluted 1:2000 served as the secondary antibody. Slides were treated with SlowFade Gold with DAPI (Invitrogen) to prevent bleaching and allow for the visualization of anatomical and cellular structures. An Olympus 1X81 FV1000 confocal microscope was used to visualize the specimens.

### Study 2a: Detection of RVFV RNA in eggs and first instar larvae of parental mosquitoes after receiving an infectious blood meal

Female mosquitoes were provided an artificial infectious blood meal (7.3 log_10_ PFU/mL) as described above (n = 50 engorged females). Fully engorged mosquitoes were housed in a carton with a cup of water and a small paper towel to allow engorged mosquitoes to deposit egg rafts in the cup. This process was repeated twice with non-infectious blood meals on day 7 and day 14 post-infectious blood meal, yielding egg rafts from three gonotrophic cycles, termed E1, E2, and E3. For this experiment, E1 egg rafts were discarded, E2 egg rafts hatched prior to collection, yielding E2 egg raft husks (n = 21) and E2 1^st^ instar larvae (30 larvae per tube, n = 48). E3 egg rafts were also collected (n = 20). All mosquito tissues were stored in 2ml screwcap tubes (Sarstedt) with 2 glass beads (Millipore) and 250μl diluent consisting of DMEM supplemented with 10% FBS, 1% Penicillin/Streptomycin, 0.1% Gentamycin, and 0.1% Amphotericin B. All samples were stored at -80°C until processing.

Prior to RT-qPCR, samples were homogenized in a MagNA Lyser (Roche) for 1 minute at 3,500 rpm and centrifuged at 12,000 x g for 1 minute. RNA was extracted from 50μl of supernatant via the MagMAX Viral RNA Isolation Kit (ThermoFisher Scientific). RT-qPCR was conducted on all RNA samples in duplicate on a QuantStudio 3 (ThermoFisher Scientific) using TaqMan Fast Virus 1-Step Master Mix (ThermoFisher Scientific). Primers used were RVFL-2912fwdgg, RVFL-2981revAC, and RVFL-2950-Probe described in Bird et al. [29]. PFU equivalents were determined by running samples with a standard curve as previously described [29].

### Study 2b: Detection of infectious RVFV particles in parental mosquitoes and progeny after receiving an infectious blood meal

**Fig 1:**
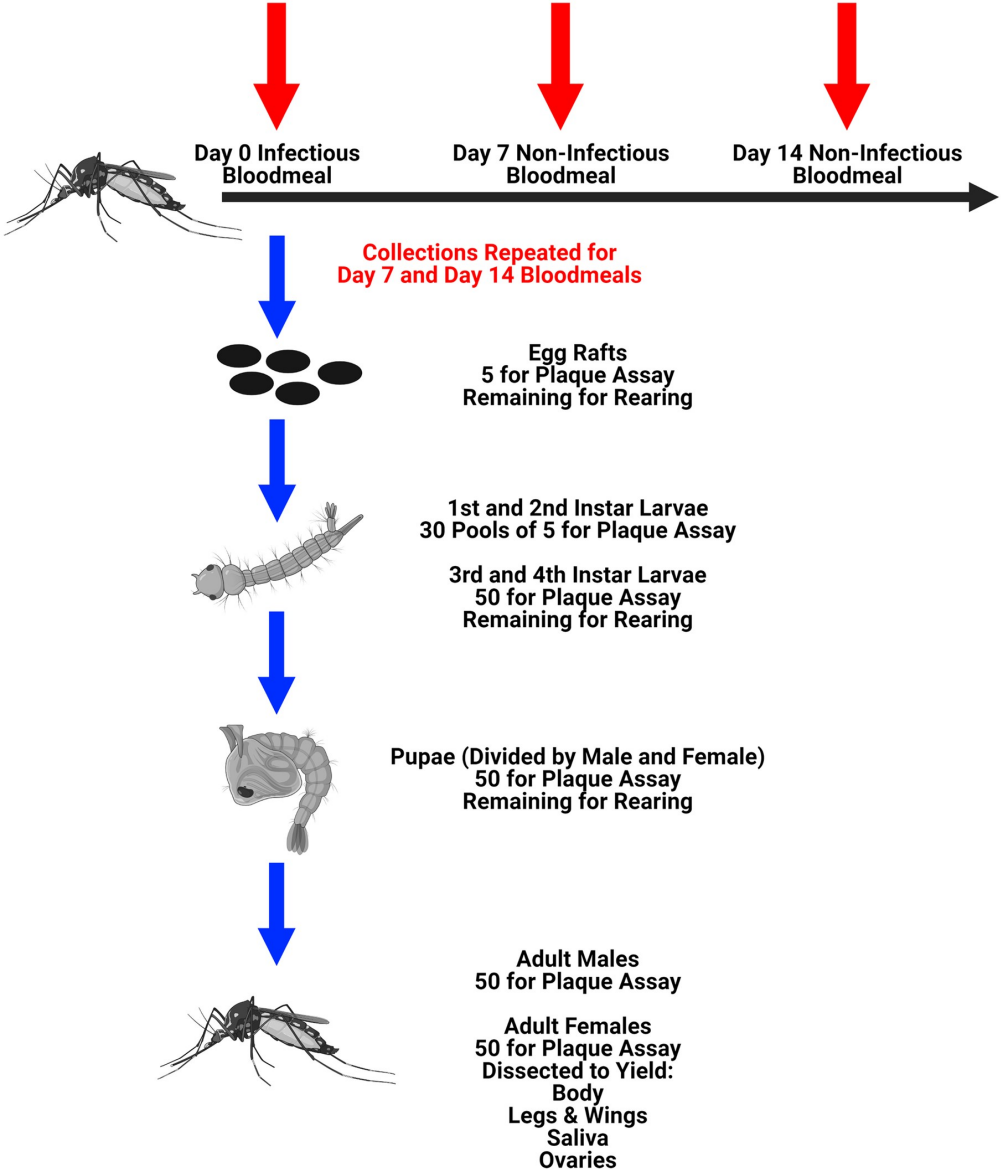
Experimental diagram for detecting the presence of RVFV among progeny mosquitoes (*Study 2b* in Materials and methods). This figure was created using BioRender.com.

Female mosquitoes were provided an artificial infectious blood meal (7.6 log_10_ PFU/mL) as described above (n = 123 engorged females). Mosquitoes were housed in a carton with a cup of water to collect egg rafts as described above. Between days 3 and 7 post-engorgement egg rafts were collected from the cup. This process was repeated twice with non-infectious blood meals on day 7 and day 14 post-infectious blood meal, yielding egg rafts from three gonotrophic cycles, termed E1, E2, and E3 (Fig 1). From each gonotrophic cycle, five rafts were collected for plaque assay and the remaining were hatched. For the first two gonotrophic cycles, the following samples were collected: pools of five first instar larvae (n = 30), pools of five second instar larvae (n = 30), individual third instar larvae (n = 50), individual fourth instar larvae (n = 50), female pupae (n = 50), male pupae (n = 50), whole male mosquitoes (n = 50), and female mosquitoes (n = 50) that were dissected to yield bodies, legs & wings, saliva, and ovaries (described below). Pupae were collected and transferred to cartons to emerge, and adult progeny mosquitoes were harvested 72 hours after the last mosquitoes emerged from their pupae. This resulted in the adult progeny having an age range of 3 to 7 days. Surviving parental mosquitoes (n = 25) were dissected on day 21 post-infectious blood meal to yield body, legs & wings, saliva, and ovaries. By the third gonotrophic cycle, death among the parental mosquitoes and a reduction in egg laying resulted in too few offspring to test all life stages; as a result, only egg rafts, adult males, and adult females were collected for analysis for the E3 progeny.

Female mosquitoes were processed in the following manner: first, legs & wings were removed, and saliva was collected as previously described [30]. After saliva collection and prior to dissection, mosquitoes were dipped in 70% ethanol immediately followed by PBS to remove hydrophobicity. Ovaries were dissected from female mosquitoes on 3x3 glass spot plates (Pyrex). All plates and tools were disinfected with 70% ethanol between samples to prevent contamination. All mosquito tissues were stored in 2ml screwcap tubes (Sarstedt) with 2 glass beads (Millipore) and 250μl diluent as described above. Saliva was stored in 1.5ml microcentrifuge tubes (Eppendorf) with 100μl diluent. All samples were stored at -80°C until processing.

Prior to plaque assays, samples were homogenized in a MagNA Lyser (Roche) for 1 minute at 3,500 rpm and centrifuged at 12,000 x g for 1 minute, except saliva which was only centrifuged, the resulting supernatant was used for plaque assays. Parental and E2 samples were titrated by 12-well plaque assay as previously described [26], using 10-fold dilutions and plating dilutions 0 through 10^−5^. Specifically, 100μl of inoculum was reserved for inoculation of the undiluted well, 1:10 dilutions (10^−1^ thru 10^−5^) were made on 96-well plates by applying 20μl of the inoculum to 180μl diluent, ensuring the inoculum was sufficiently dispersed in the diluent by mixing before transferring to the next dilution. All plaque assays were conducted in duplicate and positive titers were only recorded if both duplicates were positive for virus. Titers are reported as averages of the two duplicates. The plaque assays were designed as described above to reduce the limit of detection to the lowest level while still being able to conduct assays in duplicate and on 12-well plates; one plaque forming unit in the undiluted well (100μl inoculum) translated to 2.5 plaque forming units for the sample in question due to the 250μl diluent volume in each sample, making this our limit of detection.

Upon visualizing plaques only in the 0 dilution of the E2 samples, only undiluted samples were plaqued in single wells for virus quantification in the E1 and E3 samples. For pooled samples, namely the 1^st^ and 2^nd^ larvae, infection rates were determined by maximum likelihood estimation [31].

## Results

### Study 1: Detection of RVFV antigen in mosquitoes receiving infectious blood meal

**Fig 2:**
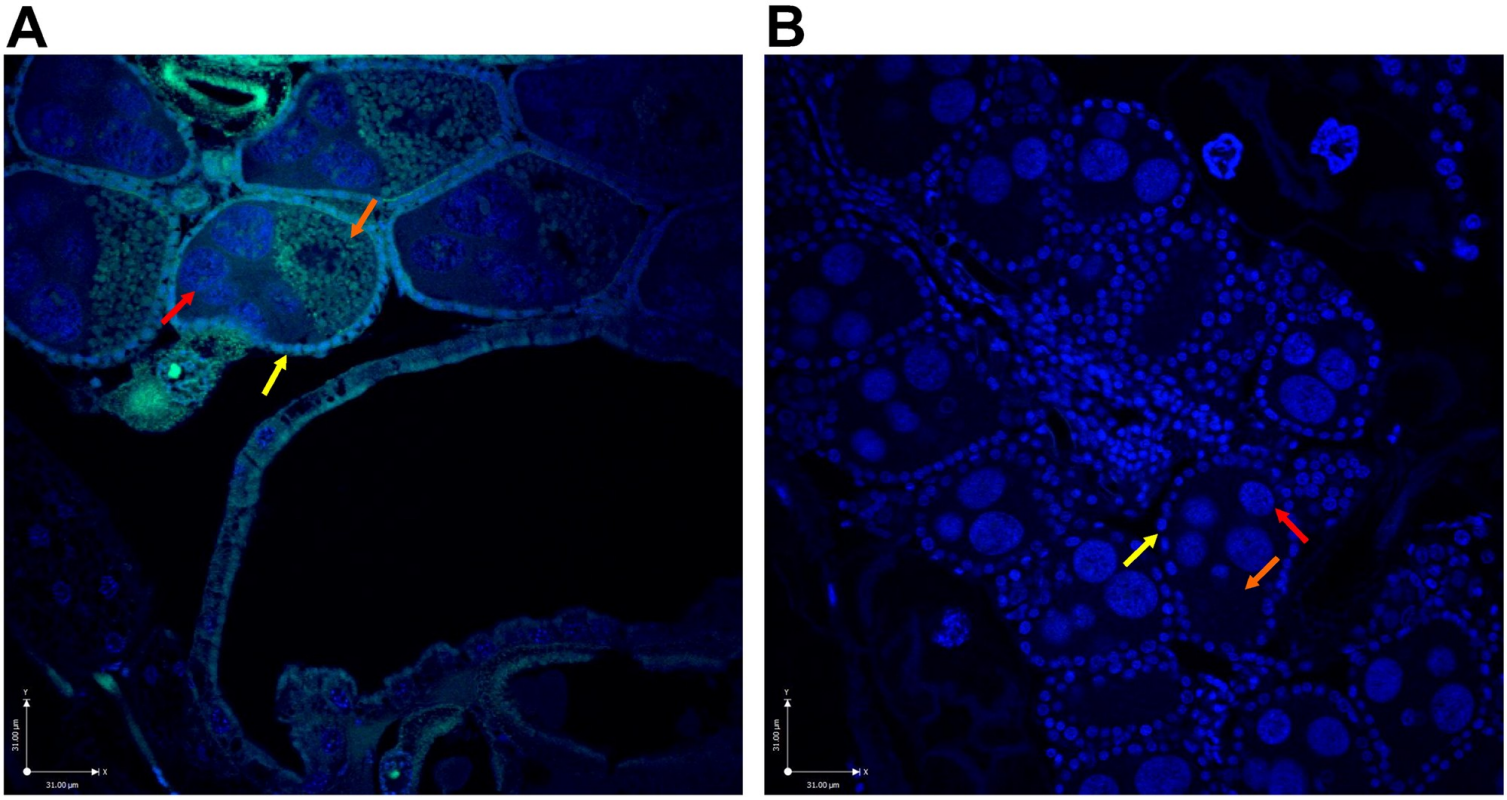
Confocal images of RVFV antigen present in ovarian tissue of RVFV-infected *Cx*. *tarsalis* (Blue = DAPI, Green = RVFV antigen). A) RVFV Infected. B) Non-infected control. Yellow arrows: follicular epithelium; red arrows: nurse cells; orange arrows: oocyte. Purple scale bar = 31μm.

**Fig 3:**
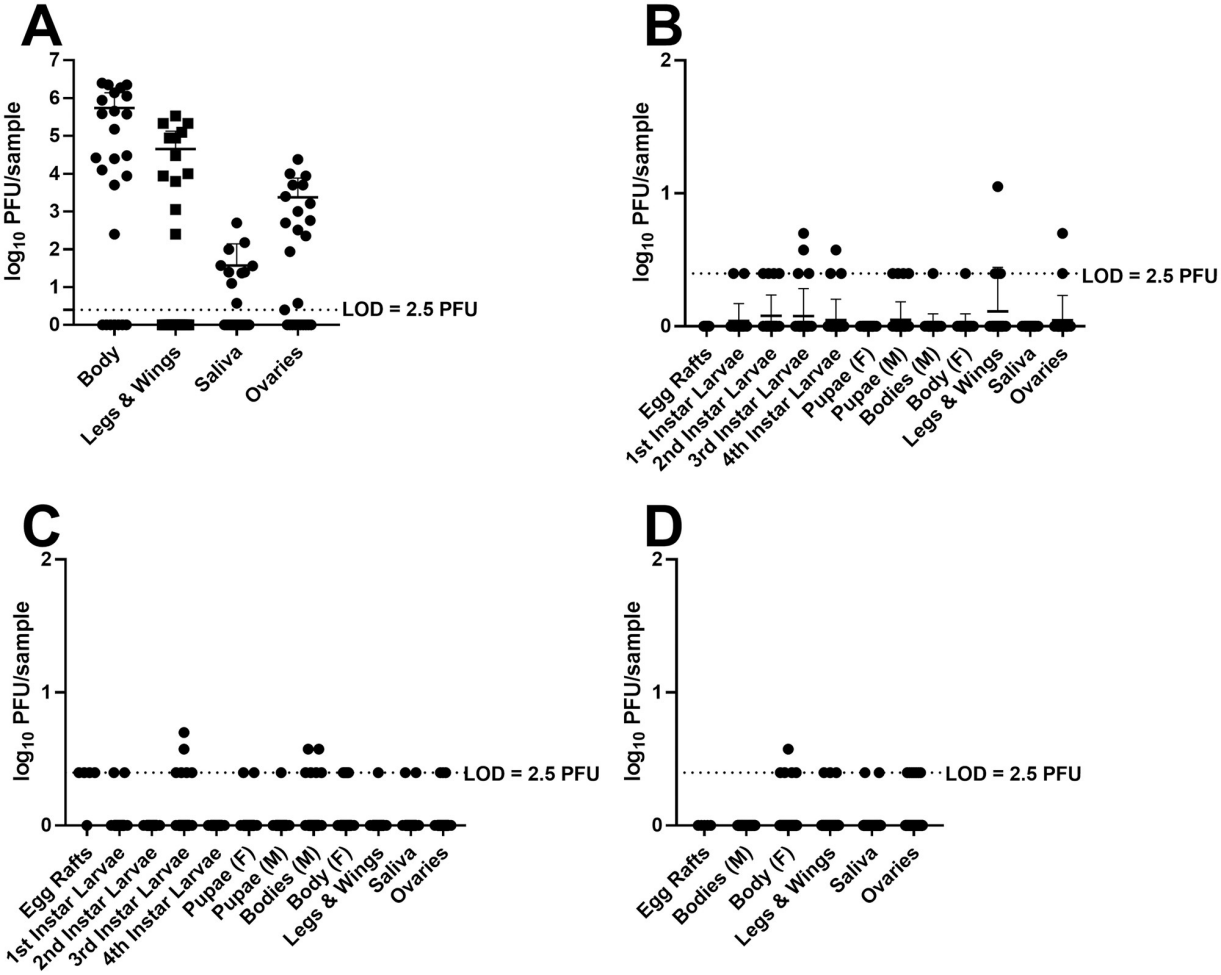
RVFV Titers of parental and progeny mosquitoes. A) Parental mosquitoes, B) E1 progeny mosquitoes, C) E2 progeny mosquitoes, D) E3 progeny mosquitoes. LOD: limit of detection for the assay.

Rift valley fever virus was administered to female *Cx*. *tarsalis* mosquitoes through an infectious blood meal delivered through an artificial membrane feeding apparatus to approximate natural exposure. RVFV antigen was identified in the follicular epithelium, oocytes, and nurse cells in the ovaries of infected parental mosquitoes by immunofluorescence assay (Fig 2). This observation was made in specimens examined after 7 days incubation. The movement of RVFV from the midgut to the ovaries was confirmed by plaque titration, demonstrating that 60% of blood-fed female mosquitoes had infectious RVFV in their ovaries at a mean virus titer of 3.4 log_10_ PFU (Table 1 and Fig 3).

**Table 1:** Percent of parental tissues testing positive for RVFV via plaque assay.

	Tested	Positive	Percent Infected†
Body	25	18	72%
Legs & Wings	25	12	48%
Saliva	25	10	40%
Ovaries	25	15	60%

### Study 2a: Detection of RVFV RNA in eggs and first instar larvae of parental mosquitoes after receiving an infectious blood meal

We then sought to determine if RVFV RNA could be detected in the eggs and first instar larvae of mosquitoes who had imbibed a RVFV bloodmeal. Parental mosquitoes were orally exposed to RVFV as above, followed by two subsequent noninfectious blood meals weekly thereafter to mimic natural blood meal acquisition and associated physiological changes. Egg raft husks and 1^st^ instar larvae from the second gonotrophic cycle (E2) and egg rafts from the third gonotrophic cycle (E3) were collected. We found that 24% of the E2 egg raft husks, 27 of 48 positive pools of 30 larvae, and 40% of the E3 egg rafts were positive for viral RNA by RT-qPCR, demonstrating the presence of RVFV RNA in the progeny of infected parental mosquitoes at early life stages (Table 2). PFU equivalents were unable to be determined on many samples from the standard curve due to the Ct values being higher than the x-intercept (33.1) of the linear regression generated from the standards, though many of the samples had Ct values < 33.1 (S1 Table). Samples listed as positive had Ct values less than the average Ct value for the most dilute sample in the standard dilution series (S2 Table). A Ct of between 30–35 has previously been shown to represent a positive result and has been associated with recovery of low levels of infectious RVFV [32,33], consistent with what we observed in our standards (S2 Table).

**Table 2:** Percent of tissues positive for RVFV RNA among progeny mosquitoes.

	F1 E2 Mosquitoes				F1 E3 Mosquitoes			
	Tested	Positive	Percent Infected†	Average Ct Value††	Tested	Positive	Percent Infected†	Average Ct Value††
Egg Rafts	0	0	N/A	N/A	20	8	40%	33.5
Egg Raft Husks	21	5	24%	33.6	0	0	N/A	N/A
1st Instar Larvae (pools of 30)	48	27	2.7%	33.7	0	0	N/A	N/A

^†^Percent infection rates for pooled samples were calculated using maximum likelihood estimation. These values are indicated with an asterisk.

^††^Average Ct values reported are the mean of the samples considered to be positive (Ct value < 35.1).

### Study 2b: Detection of infectious RVFV particles in parental mosquitoes and progeny after receiving an infectious blood meal

Next, we sought to determine whether viable RVFV was transmitted from the infected ovaries of the female to her progeny. Parental mosquitoes were orally exposed to RVFV as above, followed by two subsequent noninfectious blood meals weekly thereafter to mimic natural blood meal acquisition and associated physiological changes. Eggs from each of the three gonotrophic cycles (E1, E2, E3) were reared to the adult stage, with individuals from each developmental stage screened for infectious virus.

Vertical transmission occurred after the first infectious blood meal, as RVFV was detected in the F1-E1 progeny (Table 3 and Fig 3). Adult female progeny mosquitoes harbored infectious virus in their ovaries and saliva, indicating the potential ability to transmit vertically or horizontally upon emergence (Table 3), albeit all the titers were at or near the limit of detection (Fig 3). Third, ovary infection rates were higher than viral dissemination rates from the midgut, which may indicate a unique mechanism for the transit of RVFV from the midgut to the ovaries (Table 3). Fourth, infection rates among F1 adults appeared consistent between gonotrophic cycles (Table 3). Fifth, infectious titers were low but detectable, warranting further investigations into the physiological mechanisms for virus persistence (Fig 3). While many questions remain to be investigated, these findings are important in that they represent preliminary demonstration of vertical transmission of RVFV from a female mosquito to her progeny in a laboratory setting.

**Table 3:** Percent of tissues testing positive for RVFV via plaque assay among progeny mosquitoes.

	F1 E1 Mosquitoes			F1 E2 Mosquitoes			F1 E3 Mosquitoes		
	Tested	Positive	Percent Infected†	Tested	Positive	Percent Infected†	Tested	Positive	Percent Infected†
Egg Rafts	5	0	0%	5	4	80%	5	0	0%
1st Instar Larvae (pools of 5)	30	2	1%*	30	2	1%*	-	-	-
2nd Instar Larvae (pools of 5)	30	4	3%*	30	0	0%*	-	-	-
3rd Instar Larvae	50	4	8%	50	6	12%	-	-	-
4th Instar Larvae	50	3	6%	50	0	0%	-	-	-
Pupae-Male	50	4	8%	50	1	2%	-	-	-
Body-Male	50	1	2%	50	6	12%	50	0	0%
Pupae-Female	50	0	0%	50	2	4%	-	-	-
Body-Female	50	1	2%	50	5	10%	50	5	10%
Legs & Wings-Female	50	4	8%	50	1	2%	50	3	6%
Saliva-Female	50	0	0%	50	2	4%	50	2	4%
Ovaries-Female	50	2	4%	50	3	6%	50	7	14%

^†^Percent infection rates for pooled samples were calculated using maximum likelihood estimation. These values are indicated with an asterisk.

## Discussion

Our studies provide several lines of preliminary evidence supporting the vertical transmission of RVFV among *Cx*. *tarsalis* mosquitoes, using molecular, immunological, and virological methodologies. First, we showed that *Cx*. *tarsalis* mosquitoes, upon ingestion of an infectious blood meal, accumulated RVFV antigen in the developing oocytes of the ovaries by 7 days post exposure, evidenced by detection of antigen in the ovaries of females who received an infectious blood meal (Fig 2). Second, egg raft husks and egg rafts from the 2^nd^ and 3^rd^ gonotrophic cycles of infected mosquitoes were positive for RVFV RNA by RT-qPCR (Table 2). Third, in a separate experiment, infectious virus was detected in the tissues and saliva of progeny mosquitoes from multiple gonotrophic cycles by plaque assay (Table 3). Collectively these experiments demonstrate that RVFV is capable of vertical transmission in *Cx*. *tarsalis*, regardless of gonotrophic cycle.

The nature of this vertical transmission in *Cx*. *tarsalis* is yet to be confirmed (i.e. transovarial versus transovum), as well as if this mechanism varies by gonotrophic cycle. While viral antigen was localized to the interior of developing oocytes suggesting transovarial transmission (Fig 2), viral RNA was also detected on the husks of hatched eggs suggesting a more superficial attachment of virions (Table 2) consistent with transovum transmission [21,34]. Antigen for RVFV has previously been visualized in association with the interior and exterior of developing oocytes as well as the common and lateral oviducts and genital chamber of *Ae*. *macintoshi* mosquitoes [21]. The observations from our study involving *Cx*. *tarsalis* are consistent with this earlier work even though the mosquito species utilized represent different genera.

Additionally, we found disagreement among the tissue infections in the positive adult progeny, that is, not all mosquitoes who had virus-positive tissues had evidence of a disseminated or midgut infection (Table 3). Progeny naturally infected through vertical transmission, especially when viral antigen has been detected in the interior of the developing oocyte, would be expected to produce adult progeny with a systemic infection since the virus would have the opportunity to infect cells at an early stage of embryonic development, as opposed to needing to pass through the standard barriers to infection following oral exposure. We observed infection of both ovarian and somatic tissues in adult F1 progeny (Table 3) from parental females with viral antigen that had perfused the yolk interior of the mature oocytes (Fig 2). While we did not specifically investigate the cell types within the chorionated eggs that were infected, this result suggests that RVFV is able to infect developing cells in different poles of the oocyte that eventually differentiate into germline or somatic tissues, similarly to what has been described for *Wolbachia* [35], but this phenomenon warrants further study.

Importantly, many of our RVFV-positive samples were determined to be at the limit of detection for the plaque assay, which raises the question of false positives or contamination. We believe the independent results from the RT-qPCR data showing viral RNA in egg rafts, egg raft husks, and 1^st^ instar larvae to be compelling evidence that vertical transmission is occurring, albeit at low levels (Table 2). The threshold for positivity in these samples and recovering low levels of infectious virus was also consistent with other studies [32,33]. Furthermore, we elected to conduct plaque assays based on the RT-qPCR data to remove any uncertainty regarding the presence of infectious virus as opposed to viral RNA fragments. Samples were plaqued in duplicate, and samples with plaques in both wells were counted to add further confidence in the validity of the results. Unfortunately, to obtain low limits of detection and sample volume constraints we were unable to conduct both plaque assays and RT-qPCR on the same samples. Variability among infection rates within the same gonotrophic cycle could be due to the measured titers being at the limit of detection of the plaque assay.

There are a number of factors potentially influencing the inconsistent tissue distribution patterns observed in F1 adults as well as the low titers of RVFV in emergent progeny. One hypothesis is the influence of *Wolbachia* infection. The distribution of the intracellular bacterium *Wolbachia* varies widely by taxa and species [35] and has been known to influence West Nile virus infection of *Cx tarsalis* mosquitoes [36]. Dodson et al. also reported that while *Wolbachia* infection did not influence transmission rates of RVFV by *Culex* mosquitoes, there was a negative correlation between *Wolbachia* density and RVFV titer [37]. Romoser et al. [21] also postulated that some eggs of *Aedes macintoshi* infected with RVFV may harbor a latent infection which may go undetected during experimental investigation but is instrumental in establishing a stabilized infection in natural mosquito populations. Lastly, immune factors intrinsic to the vector such as RNA interference (RNAi) could also limit the transstadial penetration of virus through to the adult stage [38,39]. RNAi can be readily triggered in mosquito larvae following exposure to double-stranded RNA (dsRNA) [40,41]. If exogenous viral RNA present in oviposited mosquito eggs triggers RNAi in metabolically-active larvae, this natural anti-viral mechanism may influence the resulting infection rates and viral titers in F1 adults. Whether the tissue distributions and viral titers observed in this study are due to suppression by *Wolbachia*, latent infection, RNAi, or some other combination of mechanisms, this observation requires further investigation. We also found that male mosquito progeny had low rates of infection, raising the question of whether venereal transmission can further potentiate spread of the virus among mosquito populations.

Notably, there was a discrepancy between dissemination rates (48.0%) and the infection rate of the ovaries (60.0%) of parental *Cx*. *tarsalis* mosquitoes (Table 1). This phenomenon also extends to individual mosquitoes. This observed disagreement could be due to the fact that the virus titers observed were at the limit of detection and the assay was not sensitive enough to detect virus in the other tissues, and that positive detections were missed for some samples due to the low infectious titers. This observation may also be due to the virus making use of the tracheal system to disseminate throughout the mosquito body, and warrants additional exploration [42]. Romoser et al [21] localized RVFV antigen to the cytoplasm of the tracheal cells in RVFV-infected *Ae*. *macintoshi* mosquitoes and hypothesized that ovarian infections could occur through this route in addition to infection through the follicular epithelium. Kading et al. [42] also observed viral antigen in tracheal epithelial cells in *Ae*. *aegypti* mosquitoes experimentally infected with RVFV ZH501 strain. Similarly, Chandler et al. reported LaCrosse virus (LACV) infection of ovarian tissue in the mosquito *triseriatus* (Say) several days prior to virus dissemination from the midgut [43]. Utilization of the tracheal system for viral dissemination has been proposed for several other mosquito- and tick-borne viruses [44,45].

Here we report preliminary laboratory evidence for the vertical transmission of RVFV in colonized *Cx*. *tarsalis* mosquitoes for three consecutive gonotrophic cycles. These data represent important first steps towards describing the nature of this transmission mechanism and its role in RVFV environmental persistence. *Cx*. *tarsalis* is an ideal model for these preliminary experiments as the mosquito is very susceptible to infection, and would likely play a significant role in a RVFV epizootic if RVFV emerged into North America [13,16]. Furthermore, the *Cx*. *tarsalis* colony is well established and easily manipulated. Future studies will focus on validating the efficiency of vertical transmission in *Cx*. *tarsalis* and additional mosquito species, comparing virus strains, the effect of a subsequent blood meal on virus replication in F1 progeny, and understanding the tissue tropisms and immune pathways that may be involved in modulating viral replication in vertically infected *Cx*. *tarsalis*.

Demonstration of vertical transmission for RVFV in a laboratory setting has filled a research gap that has existed since the discovery decades ago that newly emerged mosquitoes can be infected with RVFV [20]. Certainly, because these experiments were conducted with a laboratory mosquito colony, the relevance of laboratory controlled vertical transmission to natural transmission cycles of RVFV in the field remains an important research aim. These data will also help parameterize models that seek to predict the establishment potential and persistence of RVFV transmission in different ecological systems, in which critical assumptions were previously necessary [46]. Still, these experiments demonstrate the proof-of-concept that RVFV, under standard laboratory conditions, can be vertically transmitted by mosquitoes.

## Supporting information

S1 Table RVFV RT-qPCR Ct values for individual mosquito F1 progeny reared from RVFV-infected parental female *Culex tarsalis* mosquitoes over three gonotrophic cycles. (XLSX)

S2 Table Standard curve for RVFV RT-qPCR assays. (XLSX)
