# Correction: Laboratory demonstration of the vertical transmission of Rift Valley fever virus by *Culex tarsalis* mosquitoes

**DOI:** 10.1371/journal.pntd.0010413

**Published:** 2022-04-26

**Authors:** Nicholas A. Bergren, Erin M. Borland, Daniel A. Hartman, Rebekah C. Kading

There is an error in Table 3. An inadvertent error in the titer calculations of progeny mosquitoes resulted in some low-titer samples being included in the analysis when they should have been counted as negative for falling below the limit of detection. The percent positive progeny in the table has been updated so that the saliva of F1 adult mosquitoes are all negative.

**Table 3:** Percent of tissues testing positive for RVFV via plaque assay among progeny mosquitoes.

	F1 E1 Mosquitoes			F1 E2 Mosquitoes			F1 E3 Mosquitoes		
	Tested	Positive	Percent Infected†	Tested	Positive	Percent Infected†	Tested	Positive	Percent Infected†
Egg Rafts	5	0	0.0%	5	1	20.0%	5	0	0.0%
1st Instar Larvae (pools of 5)	30	1	0.1%*	30	1	0.1%*	-	-	
2nd Instar Larvae (pools of 5)	30	2	1.4%*	30	0	0.0%*	-	-	
3rd Instar Larvae	50	3	6.0%	50	6	12.0%	-	-	
4th Instar Larvae	50	1	2.0%	50	0	0.0%	-	-	
Pupae-Male	50	0	0.0%	50	1	2.0%	-	-	
Body-Male	50	0	0.0%	50	6	12.0%	50	0	0.0%
Pupae-Female	50	0	0.0%	50	0	0.0%	-	-	
Body-Female	50	0	0.0%	50	2	4.0%	50	2	4.0%
Legs & Wings-Female	50	3	6.0%	50	1	2.0%	50	0	0.0%
Saliva-Female	50	0	0.0%	50	0	0.0%	50	0	0.0%
Ovaries-Female	50	2	4.0%	50	0	0.0%	50	1	2.0%

^†^Percent infection rates for pooled samples were calculated using maximum liklihood estimation. These values are indcated with an asterisk.

Fig 3 is incorrect due to the RVFV titers of progeny mosquitoes. The titers of some F1 progeny mosquitoes have been corrected for some samples, which previously fell on the LOD. These are now represented as 0. The authors have provided a corrected version here.

**Fig 3:**
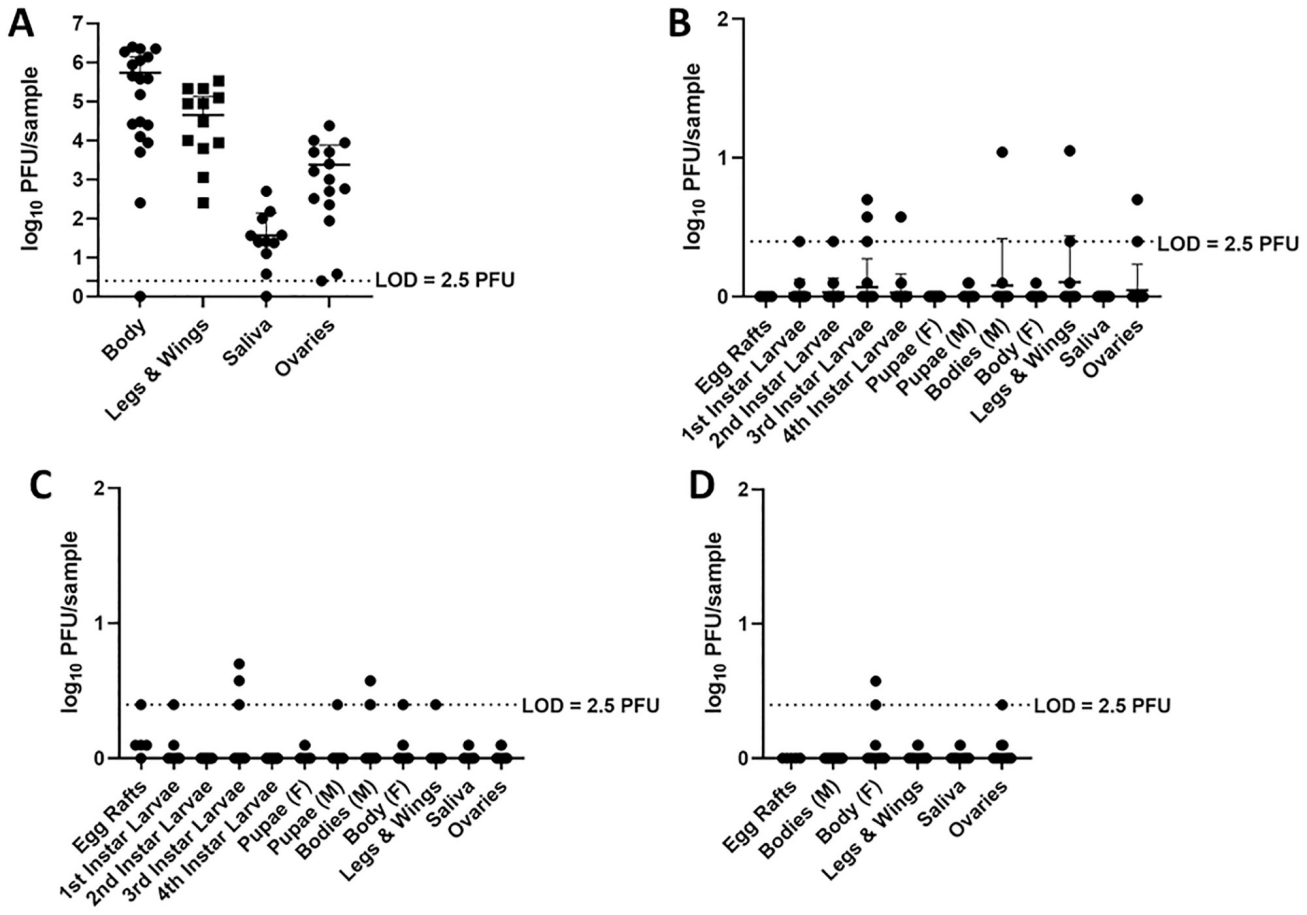


There are some minor errors in the section Results 2b and Discussion section due to the errors in the table and figure. Please see the correct Results 2b section below:

## Results, 2b

Vertical transmission occurred after the first infectious blood meal, as RVFV was detected in the F1-E1 progeny (Table 3 and Fig 3). Adult female progeny mosquitoes harbored infectious virus in their ovaries, indicating the potential ability to transmit vertically (Table 3), albeit all the titers were at or near the limit of detection (Fig 3). Third, ovary infection rates were higher than viral dissemination rates from the midgut, which may indicate a unique mechanism for the transit of RVFV from the midgut to the ovaries (Table 3). Fourth, infection rates among F1 adults generally appeared similar between gonotrophic cycles (Table 3). Fifth, infectious titers were low but detectable, warranting further investigations into the physiological mechanisms for virus persistence (Fig 3). While many questions remain to be investigated, these findings are important in that they represent preliminary demonstration of vertical transmission of RVFV from a female mosquito to her progeny in a laboratory setting.

## Discussion

Our studies provide several lines of preliminary evidence supporting the vertical transmission of RVFV among *Cx*. *tarsalis* mosquitoes, using molecular, immunological, and virological methodologies. First, we showed that *Cx*. *tarsalis* mosquitoes, upon ingestion of an infectious blood meal, accumulated RVFV antigen in the developing oocytes of the ovaries by 7 days post exposure, evidenced by detection of antigen in the ovaries of females who received an infectious blood meal (Fig 2). Second, egg raft husks and egg rafts from the 2nd and 3rd gonotrophic cycles of infected mosquitoes were positive for RVFV RNA by RT-qPCR (Table 2). Third, in a separate experiment, infectious virus was detected in the tissues of progeny mosquitoes from multiple gonotrophic cycles by plaque assay (Table 3). Collectively these experiments demonstrate that RVFV is capable of vertical transmission in *Cx*. *tarsalis*, regardless of gonotrophic cycle.
